# Association between chronic kidney disease and sarcopenia and emerging treatment strategies

**DOI:** 10.3389/fnut.2025.1699218

**Published:** 2025-11-04

**Authors:** Shuxin Li, Hongliang Cao, Zihan Gao, Yuwei Liang, Yutao Ma, Shanyu Liu, Liming Wang, Wei Wei

**Affiliations:** Department of Urology, The First Hospital of Jilin University, Changchun, China

**Keywords:** CKD, sarcopenia, chronic inflammation, oxidative stress, uremic toxin, metabolicacidosis, hormone disorders

## Abstract

Chronic kidney disease (CKD) is an irreversible and progressive kidney disease with a significant global health impact. Sarcopenia is an age-related syndrome characterized by the progressive loss of skeletal muscle mass and strength, and it exhibits a high prevalence, particularly among the elderly. There is a growing body of evidence indicating a strong bidirectional association between CKD and Sarcopenia. The prevalence of sarcopenia is significantly higher in CKD patients and increases as kidney function deteriorates. This review examines the potential relationship between CKD and sarcopenia, discusses their pathophysiological mechanisms, including chronic inflammation, oxidative stress, uremic toxin accumulation, metabolic acidosis, and hormonal disorders, and explores their clinical implications. Additionally, this review aims to elucidate potential pathogenic mechanisms and propose preventive and therapeutic strategies for CKD and sarcopenia, thereby guiding the optimization of clinical management and slowing disease progression.

## 1 Introduction

Chronic kidney disease (CKD) is a common nephrological condition with a global prevalence of approximately 10%, with women having a higher prevalence than men (1, 2). Most patients are concentrated in low- and middle-income countries, with China and India together accounting for approximately one-third of global cases (1, 3). The incidence of kidney failure is relatively low in Europe and North America. However, in recent years, with the acceleration of global population ageing and the rise in the prevalence of diabetes and obesity, the global burden of CKD has continued to rise (4–7). This not only imposes a significant economic burden on society but also severely impacts patients’ quality of life and survival outcomes. CKD refers to persistent abnormalities in kidney structure and function lasting more than three months (8). According to the classification criteria of the Kidney Disease Improving Global Outcomes (KDIGO) organization, CKD is divided into stages G1 to G5. Clinical manifestations vary significantly across different stages. Early symptoms are often subtle, with occasional hypertension, mild oedema, and increased nocturia (9, 10). In the middle stages, multi-system symptoms gradually emerge, with patients commonly experiencing generalized fatigue, loss of appetite, weight loss, gastrointestinal symptoms, and a tendency to bleed (10–12). As the disease progresses, patients may develop symptoms caused by the acarrhythmiaof metabolic waste and disruption of the internal environment, such as generalized edema due to sodium retention, arrhythmias caused by hyperkalemia, and limb weakness due to hypocalcemia (13, 14). The kidneys also play a role in the synthesis and secretion of various hormones. CKD can impair renal endocrine function, leading to disorders in glucose and lipid metabolism, as well as erectile dysfunction in males (13, 15–18). Additionally, impaired erythropoietin (EPO) synthesis and vitamin D deficiency can cause renal anemia and renal osteodystrophy (13, 18–20). When CKD progresses to end-stage renal disease, severe uremic symptoms may occur, including pericarditis, acute pulmonary oedema, severe anemia, and uremic encephalopathy (13, 19, 21, 22).

Sarcopenia is an age-related syndrome characterized by a progressive, widespread loss of skeletal muscle mass and muscle strength, accompanied by a decline in physical functions such as walking speed and balance (23, 24). The prevalence of sarcopenia varies significantly across different populations. The prevalence of sarcopenia in community-dwelling older adults ranges from approximately 10 to 27% and can exceed 50% in hospitalized patients (25, 26). Additionally, sarcopenia is an age-dependent condition, with muscle mass decreasing by 3–8% every decade after the age of 30, and the decline accelerating after the age of 70 (27–29). Furthermore, the prevalence of sarcopenia also exhibits gender and regional differences, with males typically having a higher prevalence than females, and Asian populations potentially having a higher prevalence than those in Europe and the Americas (30, 31). The primary clinical symptoms of sarcopenia include progressive muscle weakness, slowed walking speed, and recurrent falls (32–34). Severe sarcopenia can also lead to impaired physical function, reduced activities of daily living, decreased exercise tolerance, and balance disorders, which increase the risk of fractures by 2–3 times (32, 35–37). Given the established close association between CKD and the development of sarcopenia, heightened clinical vigilance for this risk in CKD patients is warranted. This article systematically reviews the epidemiological links and pathophysiological mechanisms connecting these two conditions and explores potential intervention strategies to prevent and treat sarcopenia in this vulnerable population.

## 2 Observational and experimental evidence suggest a strong association between CKD and sarcopenia

Recent observational studies have shown that the prevalence and severity of sarcopenia in patients with CKD differ significantly from those in healthy men, suggesting that CKD may play an essential role in the onset, progression, and treatment outcomes of sarcopenia.

### 2.1 The bidirectional association between CKD and sarcopenia

Accumulating evidence underscores a strong, bidirectional relationship between CKD and sarcopenia, where each condition can exacerbate the other in a vicious cycle. CKD promotes the development and progression of sarcopenia through mechanisms such as chronic inflammation, metabolic abnormalities, and the accumulation of uremic toxins (38–40). Conversely, the presence of Sarcopenia can also influence the onset and progression of CKD. Epidemiological data strongly supports this bidirectional association. A large-scale study based on the UK Biobank, involving 428,320 participants including 8,767 CKD patients, demonstrated that the prevalence of sarcopenia was significantly higher among CKD patients than in the non-CKD population (41). This association is particularly evident in disease severity. A cross-sectional study conducted by Song et al. in Shanghai revealed that among 2,213 elderly individuals, the overall prevalence of sarcopenia was 19.0%, with its incidence significantly increasing alongside the severity of CKD. Patients with renal failure were especially prone to sarcopenia accompanied by low grip strength (42). A systematic review and meta-analysis involving 42,041 patients further confirmed that nearly half of CKD patients exhibit sarcopenia, with an even higher prevalence among dialysis patients (43). It is noteworthy that this association is bidirectional. Not only does CKD increase the risk of developing sarcopenia, but sarcopenia itself is also a risk factor for new-onset CKD and accelerated decline in renal function. A cohort study of 3,676 participants aged 45 years and older without baseline CKD found that individuals with sarcopenia had a higher risk of developing new-onset CKD after 4 years of follow-up, particularly in the 60–75 age subgroup or among those with hypertension (44). Results from longitudinal studies have also shown that having sarcopenia results in a rapid decline in kidney function and an increased risk of new-onset CKD (44, 45). A Mendelian randomization study involving 7,296 participants aged 40 years and older also supports a potential causal relationship between sarcopenia or probable sarcopenia and the risk of CKD (46). In summary, healthy renal function is associated with a lower incidence of sarcopenia. In contrast, the onset of sarcopenia may accelerate the progression of CKD and serve as an essential early warning indicator for renal deterioration.

### 2.2 The coexistence of CKD and sarcopenia increases the risk of adverse outcomes

The coexistence of CKD and sarcopenia not only accelerates disease progression but also significantly increases the risk of adverse clinical outcomes in patients, including cardiovascular events, hospitalization rates, and all-cause mortality. Available epidemiologic evidence indicates a significant synergistic effect between CKD and sarcopenia, making their coexistence a critical risk factor for poor patient prognosis (41, 47–49). A prospective follow-up study involving 247 patients with end-stage renal disease found that those with sarcopenia exhibited significantly higher rates of cardiovascular disease incidence and hospitalization after five years compared to patients without sarcopenia (50). In addition, sarcopenia was associated with a significantly increased risk of death. A meta-analysis by Heitor and Ribeiro et al. (51) confirmed that declines in muscle strength, mass, and physical function markedly increase mortality risk in CKD patients, especially those on dialysis with sarcopenia. This cumulative risk effect is particularly pronounced in the cardiovascular domain. A cohort study by Jiang Lijun et al. revealed that the coexistence of CKD and sarcopenia significantly increases the risk of cardiovascular diseases such as stroke, coronary heart disease, and heart failure, as well as all-cause mortality (48). Additionally, sarcopenia is an independent risk factor for progression to end-stage renal disease and hospitalization in CKD patients. A cross-sectional study involving 8,767 CKD patients demonstrated that sarcopenia increased the risk of death by 33%, reduced the 10-year survival rate by 4%, and doubled the risk of CKD progression to end-stage renal disease (ESRD) (41). In summary, the dual burden of CKD and sarcopenia imposes significant multidimensional impacts on patients, including substantially increased all-cause mortality, elevated incidence of cardiovascular events, accelerated progression to end-stage kidney disease, and markedly diminished quality of life. To address this clinical challenge, early screening using standardized sarcopenia assessment tools should be prioritized, with particular attention to high-risk populations such as patients with advanced stages of CKD.

## 3 The main mechanisms by which CKD affects sarcopenia

**Figure 1:**
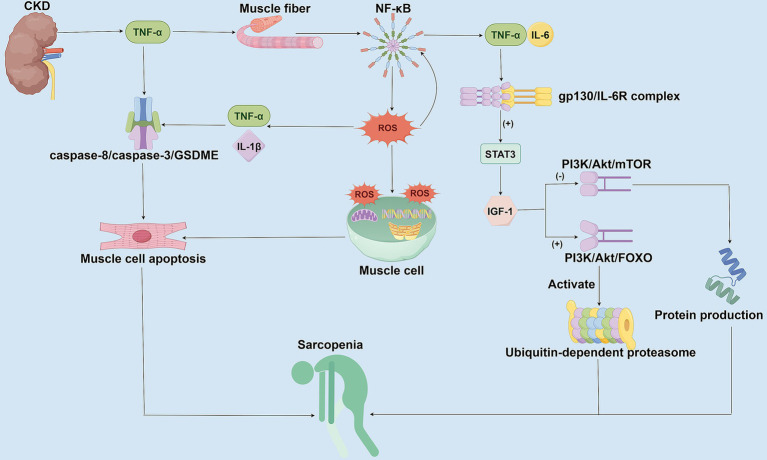
Mechanisms of chronic inflammation and oxidative stress in CKD-associated Sarcopenia. This figure illustrates the molecular mechanisms by which CKD promotes sarcopenia through chronic inflammation and oxidative stress. In CKD patients, persistently elevated inflammatory factors activate the NF-κB signaling pathway, stimulating the generation of ROS, which subsequently induces mitochondrial damage and cellular apoptosis. Concurrently, IL-6 inhibits Insulin-like Growth Factor-1 (IGF-1) function via the STAT3 signaling pathway, disrupts protein synthesis mediated by the PI3K/Akt/mTOR pathway, and enhances both the PI3K/Akt/FOXO pathway and the ubiquitin-proteasome system activity. These combined effects lead to exacerbated muscle protein degradation and myofiber atrophy.

**Figure 2:**
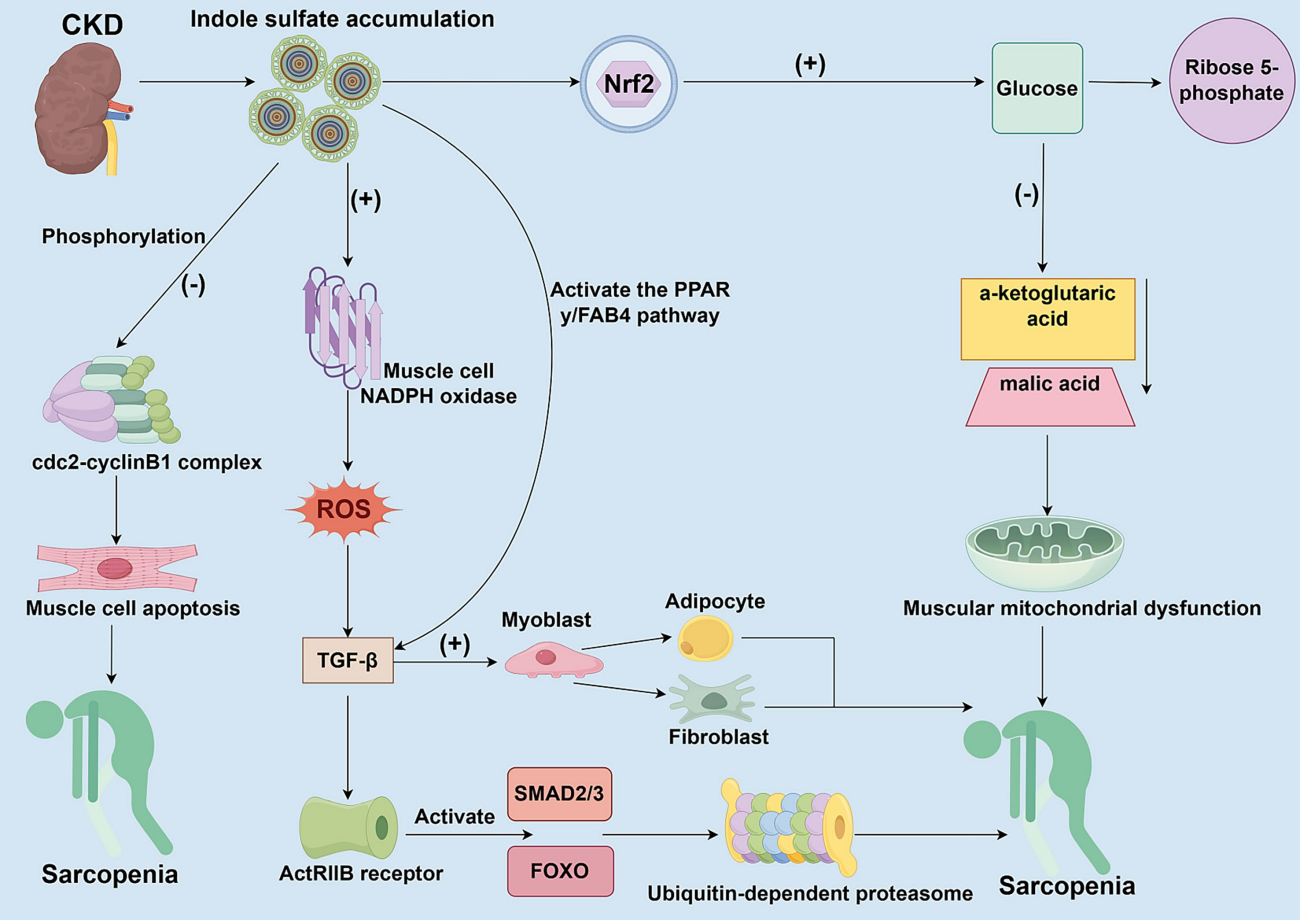
The toxic mechanisms of uremic toxins accumulate on skeletal muscle cells. This figure delineates the multifaceted detrimental effects of gut microbiota-derived uremic toxins (e.g., Indoxyl Sulfate, IS) accumulation on skeletal muscles in the context of CKD. IS activates NADPH oxidase, which increases ROS production and inhibits myoblast proliferation and differentiation. Through the ROS-ERK and JNK-MAFbx cascades, IS activates the ubiquitin-proteasome system and autophagy, promoting protein degradation. Furthermore, IS induces metabolic reprogramming via Nrf2 activation, leading to overactivation of the pentose phosphate pathway, reduced tricarboxylic acid cycle metabolites, mitochondrial dysfunction, and ATP depletion. Additionally, IS promotes fibrotic/adaptogenic trans differentiation of muscle cells via the TGF-β1/PPAR-*γ* pathway, further impairing muscle regeneration and function.

**Figure 3:**
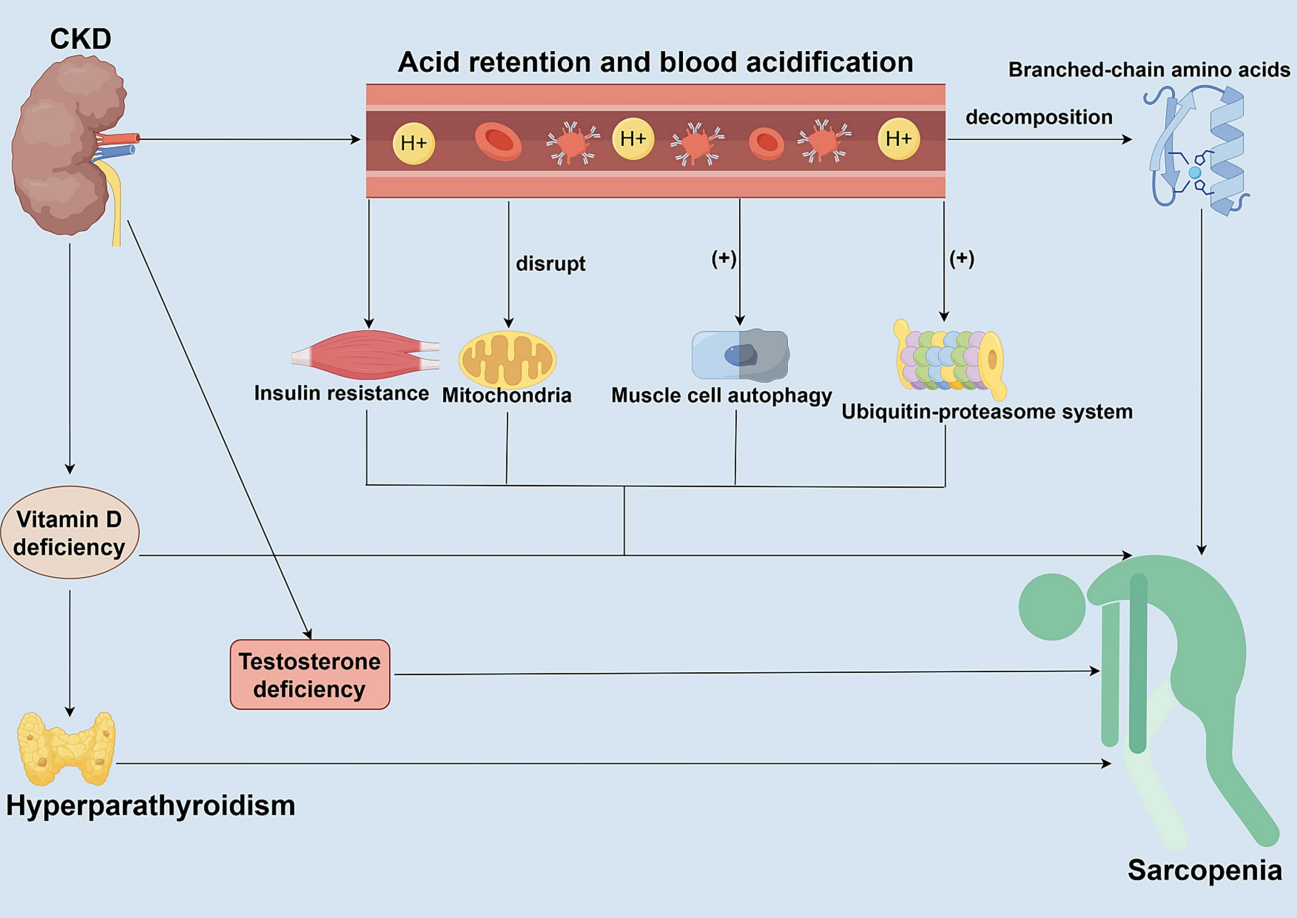
Roles of metabolic acidosis and hormonal dysregulation in CKD-associated Sarcopenia. This figure elucidates the synergistic roles of metabolic acidosis and hormonal dysregulation in driving muscle wasting in CKD patients. Metabolic acidosis directly activates the ubiquitin-proteasome system (UPS) and autophagy pathways, promoting muscle protein degradation and inducing insulin resistance. Concurrent hormonal abnormalities commonly found in CKD—such as decreased IGF-1, vitamin D deficiency, secondary hyperparathyroidism, and reduced sex hormone levels—further disrupt the balance between protein synthesis and degradation. These alterations occur through various signaling pathways, collectively exacerbating muscle atrophy.

Although current observational studies have revealed multiple associations between CKD and sarcopenia, and the differences in characterization of these studies provide targeted strategies for the prevention and treatment of sarcopenia, specific mechanisms of action need to be further elucidated. This section will elaborate on the potential mechanisms of action between CKD and Sarcopenia, as well as how CKD affects the course of Sarcopenia. There is a complex bidirectional association between CKD and sarcopenia, with mechanisms of action involving inflammatory responses, oxidative stress, toxin accumulation, metabolic disorders, and hormonal dysregulation (Figures 1**–**3).

### 3.1 Chronic inflammation and oxidative stress

CKD can contribute to the development and progression of sarcopenia through chronic inflammation and oxidative stress mechanisms (Figure 1). CKD patients are generally in a state of low-grade chronic inflammation, with persistent elevations of inflammatory markers, such as C-reactive protein (CRP), interleukin-6 (IL-6), and tumor necrosis factor-alpha (TNF-α), observed in these patients (38, 52). These inflammatory mediators promote the degradation of muscle proteins while inhibiting muscle synthesis through both direct and indirect pathways. TNF-α and IL-6 can also directly inhibit the function of muscle satellite cells, thereby impairing muscle regeneration (53, 54). When TNF-α binds to TNFR1 receptors on muscle fibers, it activates the NF-κB signaling pathway (55–57). It promotes the production of reactive oxygen species (ROS), which in turn stimulate the secretion of pro-inflammatory factors, such as IL-6 and IL-1β (55–57). ROS can damage essential components of the muscle cell, such as cellular lipids, DNA, and mitochondria, which in turn promotes the apoptosis of muscle cells (38, 55). TNF-α activates the caspase-8/caspase-3/GSDME signaling axis-mediated apoptosis via complex IIb, leading to myofiber atrophy (58). Moreover, oxidative stress-induced ROS not only exacerbate cellular damage but also continuously upregulate pro-inflammatory factors, such as IL-6 and TNF-α, through further activation of NF-κB, forming a vicious circle that amplifies inflammatory signals and ultimately leads to a progressive decline in muscle mass and function (55, 59, 60). Elevation of IL-6 also activates the STAT3 signaling pathway through the gp130/IL-6R complex, thereby inhibiting the function of insulin-like growth factor-1 (IGF-1) (59, 61, 62). In contrast, in CKD patients, reduced IGF-1 levels further impaired the PI3K/Akt/mTOR pathway-mediated protein synthesis and enhanced the PI3K/Akt/FOXO pathway, concomitant with the activation of the ubiquitin-proteasome system, which targets the degradation of muscle fibrous proteins (63–65). Therefore, chronic inflammation and oxidative stress are not only the core pathological mechanisms of CKD-associated sarcopenia but also promote each other to form a vicious cycle of inflammation-oxidative stress-muscle loss, which exacerbates the deterioration of CKD patients.

### 3.2 Uremic toxin accumulation

In patients with CKD, uremic toxins derived from the gut microbiota, such as indole sulfate (IS), accumulate in the body due to kidney clearance dysfunction. These uremic toxins can have toxic effects on skeletal muscle cells by inhibiting cell proliferation and viability (Figure 2). IS directly inhibits the proliferation and myogenic differentiation of myoblasts by activating NADPH oxidase (NOX) in myocytes, thereby increasing ROS production (66). Moreover, IS can promote muscle proteolysis and myotube atrophy by activating autophagy and the ubiquitin-proteasome system through the ROS-ERK axis and the JNK-MAFbx pathway (67). The production of ROS further triggers the release of inflammatory factors, including TNF-α, IL-6, and TGF-β1, which in turn inhibit muscle growth through chronic inflammation and oxidative stress responses (68). High doses of uremic toxin inhibit the activity of the cdc2-cyclin B1 complex through phosphorylation, block the progression of myoblasts in the G2/M phase, and induce their apoptosis, resulting in stunted skeletal muscle cell proliferation (69). Although low-dose uremic toxin does not directly inhibit myoblast proliferation, it promotes myoblast differentiation towards fibrosis and adipocytes by upregulating transforming growth factor-β1 (TGF-β1) through the activation of the PPAR-*γ*/FAB4 pathway, rather than fostering myogenic differentiation (69). In addition, members of the TGF-β family activate the SMAD2/3 and FOXO pathways by binding to the ActRIIB receptor, which synergistically promotes the degradation of muscle proteins and inhibits muscle growth, ultimately leading to muscle atrophy (59, 70). Additionally, IS accumulates in the skeletal muscle of patients with CKD, potentially leading to mitochondrial dysfunction in muscle cells. IS formed a dual effect of antioxidant and energy depletion in muscle cells through Nrf2-mediated metabolic reprogramming (71). IS enhances NADPH-dependent antioxidant defenses and increases levels of 5-phosphoribose and glutathione by activating the pentose phosphate pathway and glutathione metabolism (71). However, the long-term presence of IS can cause metabolic imbalances, overactivation of the pentose phosphate pathway, leading to the conversion of glucose shunt to 5-phosphoribose, which in turn reduces the production of key metabolites in the tricarboxylic acid cycle, such as α-ketoglutaric acid and malic acid, leading to mitochondrial dysfunction, manifested by decreased oxygen consumption rates and reduced ATP levels (71). IS can directly damage the function of the respiratory chain, which cannot be compensated for by the glycolytic pathway, eventually leading muscle cells into an energy crisis. Uremic toxin accumulation can also exacerbate muscle atrophy by interfering with the cycle and fate transformation of muscle cells, affecting muscle cell energy metabolism, and causing mitochondrial dysfunction.

### 3.3 Metabolic acidosis and hormone dysregulation

CKD significantly affects the development of sarcopenia through metabolic acidosis and hormone imbalance (Figure 3). Metabolic acidosis is one of the common complications in CKD patients, mainly caused by impaired ability of the kidneys to excrete an acid load, resulting in acid retention in the body and acidification of the blood (72). The acidic environment directly damages muscle cells and promotes the breakdown of branched-chain amino acids, thereby reducing the substrates required for muscle protein synthesis (73). Metabolic acidosis also stimulates muscle protein degradation by activating proteolytic pathways, such as the ATP-dependent ubiquitin-proteasome system (UPS) and the autophagy pathway (73, 74). Acidosis also interferes with mitochondrial function, affects metabolic processes in skeletal muscle, reduces muscle synthesis, and further exacerbates muscle wasting (74). As an anabolic hormone, insulin plays a crucial role in cellular metabolism. Insulin promotes protein synthesis and inhibits degradation through the IRS-1/PI3K/Akt pathway (73–75). However, metabolic acidosis often leads to insulin resistance in CKD patients, which reduces the sensitivity of muscle tissue to insulin (73, 74). In patients with CKD, hormone imbalances also play a crucial role in muscle metabolism. In addition to insulin resistance, patients with CKD often experience hyperparathyroidism, vitamin D deficiency, and low levels of growth hormone (GH) and insulin-like growth factor 1 (IGF-1) (76, 77). Deficiencies or impaired action of these hormones not only affect skeletal muscle synthesis but also promote muscle degradation. Low levels of GH and IGF-1 are common in CKD patients, and they are essential for muscle maintenance and repair; a deficiency of these hormones may accelerate muscle wasting (38, 77, 78). In patients with CKD, the decrease in IGF-1 levels further impairs the PI3K/Akt/mTOR pathway-mediated protein synthesis. It enhances the PI3K/Akt/FOXO pathway, simultaneously activating the ubiquitin-proteasome system and targeting the degradation of muscle fibrils (38, 77, 79). In addition, CKD can also lead to impaired vitamin D production, and vitamin D deficiency can reduce the efficiency of calcium and phosphorus metabolism, affecting calcium homeostasis and the differentiation ability of muscle cells (77, 80, 81). Vitamin D deficiency interacts with metabolic acidosis to cause endocrine changes, such as secondary hyperparathyroidism. Parathyroid hormone (PTH) promotes muscle protein breakdown by activating calcium-dependent proteases (77, 80, 81). Decreased testosterone and estrogen levels caused by CKD are also a significant risk factor for sarcopenia (77). These hormones play a key role in maintaining muscle mass and function, and hormone deficiencies and disorders can accelerate the process of muscle wasting. Correcting metabolic acidosis and modulating hormone imbalances are effective strategies to alleviate sarcopenia in patients with CKD but require a comprehensive multifactorial intervention.

### 3.4 Empirical evidence for supporting mechanism research

The molecular mechanisms of CKD-induced sarcopenia described herein are primarily derived from evidence in cellular, animal model, and clinical observational studies. To clearly illustrate the experimental basis for these findings, Table 1 summarizes key studies supporting the core pathways, including the experimental models used, the CKD stage or modeling approach, and the significant findings. These experimental designs—such as treating myotubes with uremic serum or specific toxins like indophenol sulfate and inducing CKD in mice or rats via 5/6 nephrectomy or adenine diet—provide causal evidence elucidating the roles of chronic inflammation, uremic toxins, and metabolic dysregulation in driving muscle wasting. However, it is essential to note that while these models can mimic many features of human CKD, they have limitations regarding disease progression rates, complications, and species differences. Therefore, caution should be exercised when extrapolating these mechanistic findings to CKD patients across all stages.

**Table 1:** Experimental models supporting molecular mechanisms of CKD-associated sarcopenia.

Mechanistic pathway	Key molecules/process	Experimental model	Modeling approach	Summary of key findings	References
3.1 Chronic inflammation and oxidative stress	TNF-α/NF-κB signaling pathway	Mouse	Age-related sarcopenia model	TNF-α mediates pyroptosis through the caspase-8/caspase-3/GSDME axis, leading to myofibrillar atrophy.	Wu et al. (58)
	IL-6/STAT3 signaling pathway	C2C12 Myoblasts	Recombinant IL-6 cytokine treatment	IL-6 activates STAT3, which synergizes with C/EBPδ to upregulate myostatin expression and stimulate muscle loss.。	Zhang et al. (61)
	Insulin/IGF-1 resistance (PI3K/Akt/mTOR Pathway)	Chronic renal failure rat model	5/6 Nephrectomy	CKD leads to impaired signaling in the insulin receptor substrate/PI3K/Akt pathway, which is associated with muscle atrophy.	Bailey et al. (64)
3.2 Toxin Accumulation in Uremia	IS - Induction of oxidative stress and protein degradation	C2C12 Myoblasts	IS treatment	IS increases ROS production by activating NADPH oxidase, thereby inhibiting myoblast proliferation and differentiation.	Lu et al. (66)
	IS - Activation of the proteasome and autophagy	L6 myotube cells	IS treatment	IS induces myotube atrophy through ROS-ERK and JNK-MAFbx cascades, activating the ubiquitin-proteasome system and autophagy.	Changchien (67)
	IS - Induction of mitochondrial dysfunction	C2C12 myoblasts and CKD mouse model	IS treatment and 5/6 nephrectomy + IS perfusion	IS induces metabolic reprogramming via Nrf2, leading to excessive activation of the pentose phosphate pathway and reduced tricarboxylic acid cycle metabolites, resulting in mitochondrial dysfunction and ATP depletion.	Sato et al. (71)
	Uremic toxins impair muscle regeneration.	Mouse; C2C12 myoblasts	5/6 nephrectomy; Extra-abdominal: treated with serum from uremic patients	Uremic toxins impair skeletal muscle regeneration by inhibiting myoblast proliferation, reducing myogenic differentiation, and promoting muscle fibrosis.	Alcalde-Estévez et al. (69)
3.3 Metabolic acidosis and hormonal imbalance	Metabolic acidosis - activation of the ubiquitin-proteasome System (UPS)	Rat	Chronic renal failure-induced metabolic acidosis	Metabolic acidosis promotes muscle protein degradation by activating the ATP-dependent UPS.	Ho and Abramowitz (73)
	Hormonal imbalance (IGF-1 deficiency)	Chronic renal failure rat model	Partial renal infarction	Chronic renal failure rats exhibit post-receptor defects in IGF-1 signaling within skeletal muscle, leading to impaired protein synthesis and increased degradation.	Ding et al. (65)
	Hormonal imbalance (PTH)	Mouse	Tumor-bearing mice transplanted with PTH-secreting tumors	Parathyroid hormone is a novel mediator of cachexia in experimental CKD or cancer.	Wyatt and Mitch (76)

## 4 Clinical interventions to treat and slow the progression of sarcopenia in CKD

**Figure 4:**
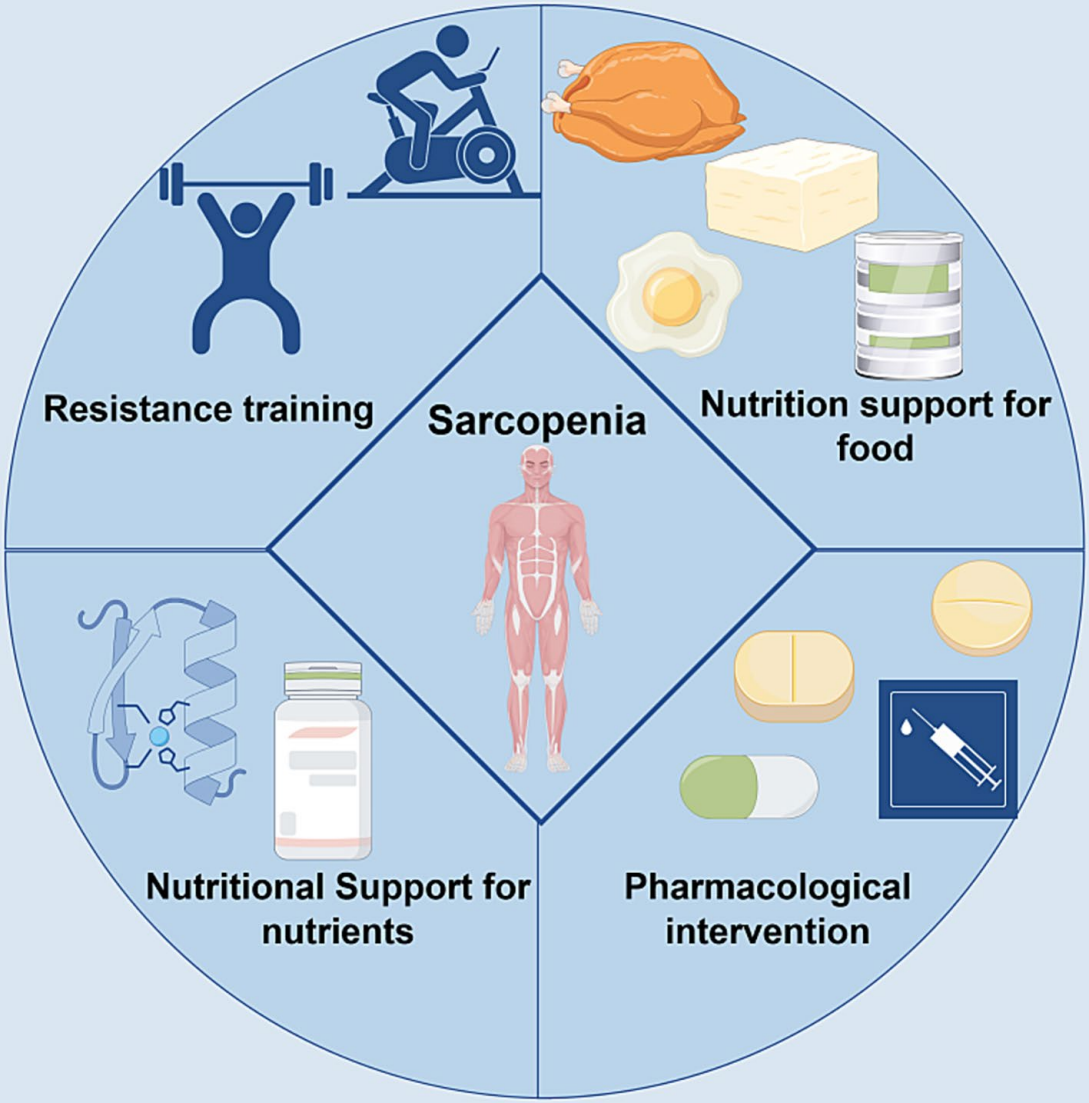
Clinical intervention strategies for Sarcopenia in CKD. Comprehensively summarizes the current clinical intervention strategies for managing Sarcopenia in patients with CKD. These multifaceted approaches target the key pathophysiological mechanisms linking CKD to muscle wasting. The interventions are primarily categorized into three pillars: exercise therapy, nutritional support, and pharmacological interventions. Exercise therapy, particularly resistance training, acts by ameliorating chronic inflammation and oxidative stress while directly stimulating muscle protein synthesis. Nutritional support, including adequate protein and branched-chain amino acid (BCAA) supplementation, addresses the anabolic resistance and protein-energy wasting (PEW) common in CKD, providing essential substrates to counteract muscle breakdown. Pharmacological strategies aim to correct specific metabolic derangements, such as using sodium bicarbonate to alleviate metabolic acidosis and testosterone replacement in hypogonadal men to stimulate anabolic pathways directly. Collectively, these strategies form a synergistic network that disrupts the vicious cycle between CKD progression and sarcopenia, highlighting the necessity of a combined and targeted therapeutic approach to improve patient outcomes.

Sarcopenia is one of the common complications in CKD patients. For sarcopenia in patients with CKD, the progression of the disease can be slowed down by a variety of interventions, including exercise, nutritional support, and medication use. These therapies play an essential role in the prevention and treatment of sarcopenia in CKD (Figure 4).

### 4.1 Sports therapy

Sarcopenia is characterized by a loss of muscle mass and strength, typically accompanied by decreased mobility and reduced endurance of exercise. CKD-related sarcopenia has become a vital complication affecting patients’ quality of life and prognosis. For Sarcopenia in CKD patients, exercise therapy is an effective way to improve muscle mass and function, as well as enhance quality of life. Recent studies have shown that exercise interventions can improve muscle metabolism and function, serving as a critical non-pharmacological means of combating sarcopenia in CKD (82). Among them, strength training, especially low-load resistance training, is efficient for CKD patients and can alleviate activity limitations due to muscle loss. In a randomized controlled trial of 53 hemodialysis patients, divided into a resistance training group (*n* = 26) and a standard exercise group (*n* = 27), a 12-week follow-up study found that resistance exercise increased muscle strength and improved muscle mass and physical function in patients with ESKD (83). Moreover, in a randomized controlled trial of 107 elderly CKD patients, 24-week pre-dialysis resistance training not only reversed the progression of sarcopenia in CKD patients but also improved the prognosis by modulating the inflammation-anemia axis, which provided a crucial evidence-based basis for the clinical implementation of pre-dialysis exercise intervention (84). In addition, a longitudinal study of 104 hemodialysis patients found that those who adhered to regular exercise experienced significant improvements in muscle strength, particularly in leg muscles. Regular exercise was an independent protective factor for muscle strength in dialysis patients, with a more pronounced effect on leg muscle strength in men of older dialysis age (≥44 months) and on handgrip strength in younger men (<44 months) (85). The study demonstrated that implementing a stabilizing exercise intervention before the onset of sarcopenia in hemodialysis patients can effectively delay dialysis-related muscle strength decline through mechanical loading and neuromuscular adaptation mechanisms. This suggests that resistance training should be prioritized in hemodialysis patients (86–88). Exercise therapy has received increasing attention as an effective means to improve sarcopenia in CKD patients. However, most current studies focus on improving muscle index, and there is a lack of long-term data on the effects of exercise interventions on adverse outcomes, such as mortality and cardiovascular events. In the future, long-term clinical outcome studies should be strengthened, and exercise prescriptions should be refined based on CKD staging and comorbidities to achieve more precise interventions.

### 4.2 Nutritional support

Nutritional support occupies a central place in the management of sarcopenia in CKD patients, specifically including protein supplementation and micronutrient use. Nutritional supportive therapy may play a role in alleviating sarcopenia in CKD patients, who are often in a protein-energy-wasting (PEW) state, a state that is a major causative factor for sarcopenia (89–91). Existing studies suggest that adequate protein and specific micronutrient intake, considering the needs and limitations of CKD patients, could help maintain muscle mass and function (75, 92). However, the evidence for the efficacy of nutritional interventions alone is less consistent than when combined with other strategies, such as exercise. In a single-center retrospective study analyzing data from patients with stage 4 CKD combined with malnutrition and comparing the effects of standard and early nutritional interventions, it was found that early nutritional interventions significantly improved muscle mass, quality of life, and nutritional status, underscoring the importance of incorporating systematic nutritional support into the management of CKD (93). Moreover, in a retrospective analysis of 260 long-term peritoneal dialysis patients, protein intake was significantly associated with changes in body composition, and lean body mass was positively correlated with protein intake (94). Maintaining a protein intake of 1.0 g/kg/d is essential to prevent muscle loss in patients with end-stage renal disease undergoing peritoneal dialysis (94). Furthermore, in a cross-sectional analysis covering 134 patients with stage 3–5 CKD, patients in the low-protein intake group demonstrated significantly lower somatic cell mass, defatted body weight, and muscle mass. Insufficient protein intake in patients with CKD was strongly associated with muscle loss, especially in females and patients with advanced disease (95). However, excessive protein intake increases renal burden, as protein metabolites such as urea and creatinine need to be excreted through the kidneys. The decreased renal excretory capacity in CKD patients may lead to exacerbation of uremic symptoms. Therefore, it is necessary to balance the protective effects of protein intake with the need for muscle maintenance in managing CKD, especially in patients already at risk for sarcopenia (96, 97). In addition, the use of nutritional drugs has provided new directions in the treatment of sarcopenia. Branched-chain amino acid (BCAA) supplementation has been shown to improve muscle protein metabolism and promote muscle synthesis, effectively slowing down muscle wasting associated with kidney disease (98–100). It is important to note that the evidence for protein or branched-chain amino acid (BCAA) supplementation as a standalone therapy remains inconclusive. While some studies, including a randomized controlled trial of 55 elderly CKD patients, showed that leucine-rich BCAA supplementation for 12 weeks significantly increased lean muscle mass, other interventions have not demonstrated clear or consistent clinical efficacy in preventing or treating sarcopenia (101). The most promising results for nutritional support often come from its combination with resistance exercise. The role of Vitamin D supplementation in managing CKD-associated sarcopenia is still under investigation. A cross-sectional study found that severe sarcopenia was significantly associated with vitamin D deficiency in patients with CKD stages 3–4 (102). Patients with vitamin D levels in the optimal range demonstrated better muscle mass and a lower prevalence of sarcopenia (102). And a prospective cohort study linked low serum vitamin D levels to an increased risk of developing sarcopenia in older men (103). In addition, a prospective cohort study that included 1,705 Australian men aged 70 years or older found that older men with low baseline serum vitamin D levels had a significantly increased risk of developing sarcopenia within 5 years (104). However, interventional trials have yet to consistently demonstrate that Vitamin D supplementation directly improves muscle mass or function in CKD patients. Maintaining adequate levels is essential for overall health, but its specific efficacy against sarcopenia requires further validation.

### 4.3 Pharmacological interventions

Pharmacological interventions for sarcopenia in CKD are an area of active research, though current evidence is limited, and the optimal strategies are not yet firmly established. Testosterone deficiency is a common problem in patients with CKD and is strongly associated with the development of sarcopenia (105). Testosterone levels in patients with CKD are significantly correlated with muscle strength, and patients with low testosterone levels are more prone to muscle atrophy and weakness (106, 107). Testosterone replacement therapy in men with CKD and confirmed hypotestosteronemia may improve muscle mass and slow the progression of sarcopenia (40, 106). However, the evidence base has limitations, and the risks and benefits must be carefully weighed for each patient. Testosterone enhances muscle mass by directly stimulating muscle growth through the activation of the androgen receptor, while inhibiting the catabolic pathway of the ubiquitin-proteasome system (105, 108, 109). CKD activates muscle protein breakdown pathways by causing metabolic acidosis, whereas the use of sodium bicarbonate reduces protein degradation by increasing the body’s pH (110, 111). Moreover, after correcting acidosis, the patient’s appetite and nutritional intake may improve, which indirectly affects muscle synthesis (111, 112). In a randomized controlled study, 42 patients with CKD stages 3–4 and metabolic acidosis were randomly assigned to a hyper bicarbonate target group and a standard group. After 4 months of intervention, a significant increase in systemic muscle mass and a decrease in muscle breakdown were observed in the hyper bicarbonate group (110). Current guidelines recommend the use of sodium bicarbonate in patients with CKD and metabolic acidosis, with a target serum bicarbonate level ≥ 22 mEq/L. However, recent studies suggest that higher target values (24–25 mEq/L) may further delay the progression of sarcopenia (110). In contrast to the more consistent benefit of correcting metabolic acidosis, the evidence for other drug classes in treating sarcopenia is less compelling. For example, interventions such as renin-angiotensin system (RAS) inhibitors,β2-adrenergic receptor agonists, and statins have not demonstrated clear or consistent clinical efficacy in preventing or treating sarcopenia in CKD patients (113–116). Although some observational studies suggest a potential association, these findings require confirmation in robust randomized controlled trials. Several novel pharmacologic agents are under investigation. Among the most promising are myostatin inhibitors, which aim to directly block a key negative regulator of muscle growth (117, 118). While still primarily in clinical trial phases for other conditions, they represent a potential future avenue for combating muscle waste in CKD. In summary, although these drug therapies show promise, most of them are still in clinical trials and have not yet been widely adopted. Pharmacotherapy needs to be combined with individualized nutritional support and exercise interventions to achieve better efficacy.

### 4.4 Interaction between clinical intervention and pathological mechanisms

The preceding sections of this review have systematically elucidated how CKD induces and exacerbates sarcopenia through core mechanisms, including chronic inflammation and oxidative stress, accumulation of uremic toxins, metabolic acidosis, and hormonal dysregulation. Current clinical intervention strategies precisely target these specific pathophysiological pathways to exert their preventive and therapeutic effects. These interventions do not operate in isolation but interact with key molecular pathways to form a multi-target therapeutic network. Exercise therapy, particularly resistance training, derives part of its benefits from regulating chronic inflammation and oxidative stress. Regular exercise has been shown to reduce serum levels of pro-inflammatory cytokines such as TNF-α and IL-6 in CKD patients (83, 84). This directly attenuates the activation of NF-κB and STAT3 signaling pathways by these factors, thereby reducing their suppression of insulin-like growth factor-1 function and downregulating ubiquitin-proteasome system-mediated muscle protein degradation (55, 59, 64). Concurrently, exercise alleviates oxidative stress-induced damage to myocytes by improving mitochondrial function and reducing excessive ROS production, thereby breaking the vicious cycle of inflammation-oxidative stress-muscle loss (38, 55, 60). And nutritional support strategies directly address the anabolic dysfunction caused by uremic toxin accumulation and PEW. Adequate supplementation of protein and BCAAs provides essential substrates for muscle protein synthesis, which is crucial in counteracting uremic toxins such as indole-3-carbinol sulfate (I3CS). IS promotes muscle breakdown by activating pathways like ROS-ERK/JNK-MAFbx, enhancing autophagy and ubiquitin-proteasome system activity (66, 67). Adequate nutritional intake counteracts this catabolic process by promoting protein synthesis mediated through the PI3K/Akt/mTOR pathway (75, 98). Furthermore, vitamin D supplementation not only corrects CKD-associated vitamin D deficiency but may also indirectly counteract uremic toxin-induced myocyte fibrotic/adaptogenic trans differentiation by improving intracellular calcium homeostasis and differentiation capacity, thereby preserving muscle mass and function (77, 80, 102). Finally, the core of pharmacological intervention lies in correcting metabolic acidosis and hormonal imbalances—key pathogenic mechanisms. Correcting metabolic acidosis with sodium bicarbonate directly inhibits the activation of UPS and autophagy pathways by an acidic environment, reduces branched-chain amino acid breakdown, and creates a favorable internal environment for muscle synthesis (110, 111). For male CKD patients with overt hypogonadism, testosterone replacement therapy directly stimulates muscle growth by activating androgen receptors. It inhibits catabolic pathways via the ubiquitin-proteasome system, thereby counteracting muscle wasting caused by testosterone deficiency commonly observed in CKD progression (105, 108). Although direct evidence for agents like RAS inhibitors, statins, and β2-adrenergic agonists in sarcopenia remains inconsistent, their potential benefits may relate to anti-inflammatory and antioxidant properties that indirectly influence these pathways (113, 116). In summary, clinical management strategies for CKD-associated sarcopenia are intricately intertwined with its underlying molecular mechanisms. Exercise, nutrition, and pharmacological interventions collectively form a synergistic network. Through multi-targeted approaches, they aim to disrupt the vicious cycle of mutual reinforcement between CKD and sarcopenia, providing a robust theoretical foundation and practical direction for improving patient outcomes.

### 4.5 Limitations and challenges in clinical research related to interventions

Although the studies mentioned above provide valuable insights for interventions targeting sarcopenia in CKD patients, existing clinical evidence exhibits significant heterogeneity and limitations. Caution is warranted when interpreting results and formulating generalizable recommendations. Variations across studies often arise from the demographic characteristics of study populations, methods for assessing sarcopenia, specific intervention protocols, and overall research quality. Table 2 systematically summarizes the primary limitations of key clinical studies mentioned in this section, including sample size, follow-up duration, dropout rates, control group design, statistical adjustments, and control of important confounders such as comorbidities and medication use. Common challenges include: insufficient statistical power due to small sample sizes; lack of long-term follow-up to assess effects on hard endpoints like mortality and cardiovascular events; difficulties in blinding and adherence issues in exercise studies; challenges in precisely controlling dietary intake and balancing protein supplementation with renal protection in nutrition studies; and the scarcity of large-scale, multicenter randomized controlled trial (RCT) evidence in pharmacologic studies. Acknowledging these limitations is crucial for accurately interpreting the strength of existing evidence, guiding future study designs, and achieving personalized clinical management.

**Table 2:** Major limitations and controversies in clinical intervention studies for CKD-associated sarcopenia.

Intervention category	Representative studies	Key limitations and points of controversy
Exercise therapy	Chaovarin et al. (83)	Small sample size (*n* = 53); single-center study in hemodialysis patients. Lack of blinding and potential for placebo effect. Dropout rates are not reported. Short follow-up (12 weeks) lacks data on long-term hard endpoints.
	Gadelha et al. (84)	The study population consisted of community-dwelling elderly (≥60 years) CKD patients; results may not apply to younger or frailer cohorts. Although improvements in the inflammation-anemia axis were demonstrated, statistical adjustment for all potential confounders may have been inadequate.
Nutritional support	Sunsandee et al. (101)	This RCT demonstrated the benefits of BCAAs, yet it contradicts other studies reporting adverse outcomes, suggesting that optimal dosage, target populations, and treatment duration remain unclear. Protein/BCAA supplementation requires balancing muscle maintenance with avoiding exacerbation of uremia. This equilibrium point varies according to CKD stage and nutritional status, making it difficult to establish a universal protocol.
	Barril et al. 2018 (95)	This study is a cross-sectional analysis and can only demonstrate associations, not establish causation. Although an association between protein intake and muscle mass was observed, residual confounders—such as inflammation levels, physical activity, and non-renal comorbidities—may influence the results.
Vitamin D supplementation	Multiple studies have shown (102–104)	Observational studies consistently show an association between vitamin D levels and sarcopenia, but interventional trials have failed to consistently demonstrate that vitamin D supplementation directly improves muscle mass or function. This suggests that low vitamin D may serve as a marker of disease severity rather than a reversible cause.
Pharmacological intervention	Bicarbonate use (110)	The sample size was small (*n* = 42), and the study was single center. The primary outcome was change in muscle mass, which was a surrogate endpoint rather than functional improvement or a clinical hard endpoint.
	Testosterone replacement therapy (106, 107)	Evidence primarily stems from small-scale, short-term studies. Insufficient research on long-term CV and other safety risks in CKD populations limits its widespread application. Benefits are confined to male patients with confirmed hypogonadism and lack universal applicability.
	RAS inhibitors/statins (113–115)	Most evidence stems from observational studies or post-hoc analyses of CV outcome trials, which are subject to uncontrollable confounding biases. Evidence from RCTs specifically targeting sarcopenia prevention/treatment is either lacking or negative.

RCT, randomized controlled trial; CKD, chronic kidney disease; CV, cardiovascular.

## 5 Conclusion and future directions

There is a complex bidirectional association between CKD and sarcopenia, which interacts in a vicious cycle through multiple mechanisms, including chronic inflammation, oxidative stress, uremic toxin accumulation, protein energy depletion, metabolic acidosis, and hormonal imbalance. This coexistence not only accelerates disease progression but also significantly increases the risk of cardiovascular events, all-cause mortality, and ESRD. Current intervention strategies vary in their level of evidence. The most consistent benefits to date have been observed with resistance exercise combined with nutritional support, correction of metabolic acidosis, and, in some studies, the use of androgens. The efficacy of other interventions, including vitamin D, protein supplementation alone, RAS inhibitors, and statins, remains less clearly established. Future research directions should focus on delving into the specific molecular mechanisms underlying CKD and sarcopenia, especially the regulation of mitochondrial dysfunction and autophagy pathways. In addition, individualized comprehensive management protocols tailored to the CKD stage, patient age, and co-morbid conditions should be developed and combined with multi-omics techniques to predict the risk of muscle loss, thereby guiding the development of effective treatment plans. Large-scale prospective studies on the effects of exercise and nutritional interventions on long-term survival, cardiovascular event rates, and quality of life in CKD patients should also be strengthened in the future. At the same time, resources from multiple disciplines, including nephrology, geriatrics, rehabilitation medicine, and nutrition, should be integrated to establish standardized assessment tools and early screening systems, thereby improving the overall prognosis for patients with CKD.
